# Retrograde longitudinal imaging analyses of *IDH*-wildtype glioblastoma reveal its clinical timeline from radiological birth to death

**DOI:** 10.1093/noajnl/vdaf275

**Published:** 2026-01-02

**Authors:** Ryota Shigeeda, Ichiyo Shibahara, Yasushi Orihashi, Yoko Tanihata, Kazuko Fujitani, Mariko Toyoda, Yuri Hyakutake, Hajime Handa, Madoka Inukai, Sumito Sato, Mitsuhiro Shinoda, Hideto Komai, Kohei Uemasu, Takashi Kiga, Hiroyuki Koizumi, Daisuke Yamamoto, Kazuhiro Miyasaka, Tomoko Sekiguchi, Chihiro Matsumoto, Mari Kusumi, Hidehiro Oka, Takuichiro Hide, Toshihiro Kumabe

**Affiliations:** Department of Neurosurgery, Kitasato University School of Medicine, Sagamihara, Japan; Department of Neurosurgery, Kitasato University School of Medicine, Sagamihara, Japan; Clinical Research Center in Hiroshima, Hiroshima University Hospital, Hiroshima, Japan; Department of Neurosurgery, Kitasato University School of Medicine, Sagamihara, Japan; Gene Analysis Center, Kitasato University School of Medicine, Sagamihara, Japan; Department of Neurosurgery, Kitasato University School of Medicine, Sagamihara, Japan; Department of Neurosurgery, Kitasato University School of Medicine, Sagamihara, Japan; Department of Neurosurgery, Kitasato University School of Medicine, Sagamihara, Japan; Department of Neurosurgery, Kitasato University School of Medicine, Sagamihara, Japan; Department of Neurosurgery, Kitasato University School of Medicine, Sagamihara, Japan; Department of Neurosurgery, Kitasato University School of Medicine, Sagamihara, Japan; Department of Neurosurgery, Kitasato University School of Medicine, Sagamihara, Japan; Department of Neurosurgery, Kitasato University School of Medicine, Sagamihara, Japan; Department of Neurosurgery, Kitasato University School of Medicine, Sagamihara, Japan; Department of Neurosurgery, Kitasato University School of Medicine, Sagamihara, Japan; Department of Neurosurgery, Kitasato University School of Medicine, Sagamihara, Japan; Department of Neurosurgery, Kitasato University School of Medicine, Sagamihara, Japan; Department of Neurosurgery, Kitasato University School of Medicine, Sagamihara, Japan; Department of Neurosurgery, Kitasato University School of Medicine, Sagamihara, Japan; Department of Neurosurgery, Kitasato University Medical Center, Kitamoto, Japan; Department of Neurosurgery, Kitasato University Medical Center, Kitamoto, Japan; Department of Neurosurgery, Kitasato University School of Medicine, Sagamihara, Japan; Department of Neurosurgery, Kitasato University School of Medicine, Sagamihara, Japan

**Keywords:** early-stage, glioblastoma, low-grade glioma, radiological birth

## Abstract

**Background:**

Glioblastoma (GB), *IDH*-wildtype, and low-grade glioma appear indistinguishable in their early pre-symptomatic phase, yet GB follows a far more aggressive clinical course. While genomic studies suggest a “biological birth” of GB years before diagnosis, when GB first becomes radiologically detectable (radiological birth) remains unknown.

**Methods:**

We analyzed longitudinal imaging data from 67 early-stage glioblastoma (earlyGB) cases, characterized by small, asymptomatic lesions that later progressed to classic magnetic resonance imaging appearance of GB (classicGB), comprising 44 institutional and 23 from published reports. A mathematical model integrating tumor volume, radius, imaging intervals, clinical data, and molecular features estimated radiological birth and its modifiers.

**Results:**

The median interval from earlyGB to classicGB was 155 days (range: 35-1557) in our cohort and 113 days (range: 4-854) in the published cohort. Radiological birth occurred 0.83 years (95% CI: 0.66-1.10) before diagnosis in our cohort and 0.15-0.92 years in the published cohort. Rapid progression correlated with age <65 years, MIB1 labeling index ≥30%, and copy-number alterations (CNAs) in *EGFR*, *PTEN*, or *CDKN2A*, but not with *TERT* promoter status. Absence of these CNAs prolonged the radiological birth to 2.27 years (95% CI: 0.79-100), indicating slower progression. Median overall survival of our cohort was 1.7 years, yielding a radiological-birth-to-death span of 2.8 years.

**Conclusions:**

This largest earlyGB cohort defines the radiological birth and entire clinical trajectory of *IDH*-wildtype GB. These findings bridge the gap between biological and radiological birth and offer a benchmark for surveillance and early-intervention strategies.

Key Points

- Longitudinal imaging data from early-stage glioblastoma can estimate its radiological birth.
- Early-stage glioblastoma and incidental low-grade gliomas are radiologically indistinguishable in the early phase, yet their prognosis differs markedly.

Importance of the Study

Early-stage glioblastoma (earlyGB), a small, non-enhancing, asymptomatic radiological lesion that later transform into lethal glioblastoma (GB), is encountered rarely in clinical practice and is usually reported as case studies. Taking advantage of Japan’s high magnetic resonance imaging (MRI) utilization documented by Organization for Economic Co-operation and Development data, we assembled the largest longitudinal earlyGB cohort. By integrating serial volumetric data with clinical and molecular profiles, we can track GB from its radiological birth to death, bridging the gap between the biological birth suggested by genomic data and radiological birth. This unique dataset establishes when GB initially appears on MRI. Furthermore, we translate the notion of earlyGB into incidental low-grade gliomas (iLGG) because both are radiologically indistinguishable in the early phase of glioma. Early surgery and watch-and-wait strategies are the debate in iLGG. Our findings provide the outcome if earlyGB does not undergo early surgery and only undergoes surgery after classical GB appears, guiding risk-adapted management strategies for aggressive glioma.

Gliomas, including glioblastoma (GB), are thought to progress through sequential stages, such as biological birth, occult phase, radiological birth, silent phase, symptomatic phase, progression, and ultimately death.^1^ Whole-genome sequencing of matched primary and recurrent *IDH*-wildtype GB indicates that biological birth precedes GB diagnosis by 2-7 years,^2^ implying a prolonged occult period during which the tumor is radiologically invisible. Estimating when the lesion first becomes radiologically detectable—the radiological birth—is therefore essential for understanding the clinical timeline of GB.

While most gliomas are radiologically detected during the symptomatic phase, we occasionally encounter rare, asymptomatic, nonspecific, non-enhancing lesions on magnetic resonance imaging (MRI) during a clinically silent phase. At this early stage, it is uncertain whether the lesion will remain as low-grade gliomas (LGG) or later progress to GB. Lesions that stay LGG are termed incidental low-grade gliomas (iLGG), with an incidence of approximately 0.025%-0.3% in the general population.^3^^,^^4^ Conversely, non-enhancing lesions that later develop into classic MRI features of GB (classicGB) are referred to as early-stage glioblastoma (earlyGB).^5-8^ Across cohorts of iLGG; 14.7%-31% harbored *IDH*-wildtype on molecular testing,^9-11^ indicating that a subset of these non-enhancing lesions ultimately classified as GB under WHO 2021 classification. Retrospective studies suggest that early surgery for iLGG yields comparable overall survival (OS) irrespective of *IDH* mutation status.^9^^,^^11^ Thus, early intervention for iLGG may alter the course of a subset otherwise destined to progress to GB. Nevertheless, management remains controversial because the natural history of incidentally detected non-enhancing lesions—particularly those that evolve into GB—is poorly documented, sustaining the debate between early surgery and watch-and-wait strategies.iLGG typically grows slowly^1^^,^^12^ and can be tracked with serial radiological imaging. In contrast, the natural progression of earlyGB remains unexplored, as GB often necessitates early surgical intervention once radiological findings suggest its presence. Prior studies of GB growth dynamics^5^^,^^6^^,^^8^^,^^13-32^ and radiological birth^33^ have relied on 2 or more short-interval scans of untreated or presurgical GB. Although such MRI data are easy to obtain, they primarily capture the aggressive growth phase of GB and offer limited insight into the transition from radiological birth to earlyGB, and ultimately to classicGB. The concept of earlyGB is important because MRI findings during the silent phase represent the earliest radiological timepoint to the radiological birth of GB. Analyzing longitudinal MRI data from earlyGB provide a unique opportunity to investigate the early phase of GB; however, such analyses remain unexplored.

In this study, we assembled the largest longitudinal earlyGB cohort to date. By integrating serial volumetric imaging with clinical and molecular data, we estimate the radiological birth of GB, characterize factors that accelerate or delay its growth, and provide evidence of potential intervention windows. These data will bridge the gap between biological and radiological birth, refine surveillance protocols, and guide risk-adapted management for aggressive glioma.

## Methods

### Patient Cohorts

This study is a single-center retrospective study approved by the ethics committee of Kitasato University School of Medicine (IRB: B21-245). We reviewed all patients histologically diagnosed with GB between July 1997 and January 2023, totaling 312 patients. The definition and inclusion criteria for classicGB and earlyGB were as follows.

The inclusion criteria for classicGB, defined as the classic appearance of GBM on MRI, were based on previously published reports.^6^^,^^8^^,^^34^ These criteria included:

1. heterogeneous cystic or necrotic enhancement on gadolinium-enhanced T1-weighted MRI (T1Gd); and at least one of the following supporting features:
2. hyperintense regions extending beyond the enhancing lesion, corresponding to peritumoral edema;
3. the presence of small intratumoral hemorrhagic components; and
4. regions demonstrating hyperperfusion when perfusion imaging was available.

The inclusion criteria for the earlyGB were:

1. histologically confirmed GB cases that underwent surgery at classicGB stage; and
2. availability of prior radiological studies, either MRI or computed tomography (CT), obtained before the detection of the classicGB on MRI.

We extracted 2 cohorts from this dataset, the earlyGB cohort and the short-interval cohort. The earlyGB cohort comprised all available imaging before classicGB detection, though some cases were not optimal for accurately estimating radiological birth. Because of this inclusion, potential distortion could arise when the intervals between imaging studies were excessively long. Specifically, when the interval between the initial image with no measurable tumor and a subsequent image demonstrating classicGB is prolonged, the estimated radiological birth may be ­substantially inaccurate. For example, an MRI taken 10 years prior to classicGB that showed no measurable tumor could yield a misleading estimate of radiological birth.

Based on previous genetic analyses, the biological birth of GB ranges from 2-7 years before diagnosis.^2^ Since the radiological birth of GB cannot precede biological birth, we excluded patients whose interval between the first radiological study, which revealed no detectable lesion, and the subsequent image exceeded 2 years. We selected the 2-year threshold because using the broader 2-7 years range would create a potential overlap period during which genetic alterations may already exist despite the absence of radiological detection. Overall, earlyGB cohort included cases with radiologically detectable lesions before the detection of classicGB and cases with no detectable lesions within 2 years before the detection of classicGB. As a result, 44 patients remained and were referred to as the earlyGB cohort. For reference, we also created an additional cohort without applying 2-year threshold, which included all earlyGB cases and comprised 66 cases (defined as earlyGB-all cohort).

For the comparison cohort, we included GB cases that underwent 2 or more MRI scans of untreated or presurgical GB, as commonly used in previous studies.^5^^,^^6^^,^^8^^,^^13-32^ Since these images were taken at relatively short intervals, we defined this as the short-interval cohort, comprising a total of 51 patients. In both cohorts, patient demographics and MIB1 labeling index (LI) were obtained from medical records.

To support our earlyGB cohort, we reviewed previous studies focusing on earlyGB (Supplementary Table S1).^5^^,^^6^^,^^8^^,^^13-23^^,^^25^^,^^27^^,^^29^^,^^31^^,^^32^^,^^35-37^ Among these, 4 studies^8^^,^^21^^,^^23^^,^^29^ including 23 cases harbored detailed data on tumor volume and the interval between radiological studies, equivalent to our earlyGB-cohort. We defined these cases as other-earlyGB cohort.

### Preoperative Tumor Volumetric Analysis

We performed serial assessments of tumor volumes using DICOM software. We used MRI or CT images with a slice thickness of 5 mm or less for volumetric analysis. For earlyGB stage, FLAIR or T2-weighted images (T2WI) are preferred to calculate non-enhancing lesions. However, because earlyGB imaging is extremely rare and the initial imaging was often performed at other hospitals before referral to our institution, available sequences varied across institutions and time points. To maximize case inclusion, we included all available pre-diagnostic modalities. When MRI was unavailable and CT was the only imaging modality at the early phase, CT was used for volumetric analysis; however, this was uncommon, occurring in only 6 of the 44 cases in earlyGB cohort. For classicGB stage, tumor volumes were delineated using T1Gd in combination with FLAIR or T2WI. Regions of interest were manually segmented around the borders of the tumor to calculate tumor volumes (cm^3^) by Neuronavigation system (BrainLab AG, Munich, Germany). All measurements were performed by 2 qualified neurosurgeons experienced in glioma surgery, who were blinded to the clinical data.

### Analysis of the Growth Model

The tumor volume at the radiological birth was defined as the minimal measurable voxel size of 0.001 cm^3^. To analyze tumor growth, the radius of each tumor modeled as a sphere was calculated from the tumor volume. Then, we applied a linear mixed-effects model using either an exponential growth model or a linear radial growth mode.^24^ The aim of the analysis is the retrograde estimation of radiological birth from the classicGB detection as day 0; thus, we did not use a Gompertzian growth model. This model is featured by a declining growth rate at one point during the later phase of the tumor growth,^24^ which is not the interest of current study. Also, a previous report showed the tumor growth at early phase was identical among linear radial, exponential and Gompertzian growth models. In particular, the exponential growth tumor models presented slower tumor growth in cases with small tumor (1 mL).^24^ The selection between these models was determined based on a visual assessment of the normality for the random effects and residuals, the plausibility of the “radiological birth” estimates, and marginal and conditional *R*^2^ values.^38^

In our analysis, 0 on the Y-axis represented the point of radiological birth. Interactions between time and the variables were included as fixed effects. Radiological birth was estimated as the time point at which the tumor volume would reach 0 (*Y* = 0 on the Y-axis), calculated using the fixed-effect estimates (slope and intercept) from the mixed-effects model. This value is not a summary statistic of the patient-specific radiological birth, which would require incorporating both fixed and random effects. The 95% confidence interval (CI) was calculated using Fieller’s theorem based on the standard errors of the slope and intercept. Therefore, the radiological birth obtained from the fixed effects should be interpreted as a model-predicted typical value for the cohort rather than an average of the individual patient-specific estimates. The analyses of the growth model were performed using SAS 9.4 (SAS Institute Inc., Cary, North Carolina, United States).

### Molecular Analyses

The tumor tissue specimens were stored at −80 °C until the extraction of DNA. Genomic DNA was extracted from fresh frozen specimens using the QIAamp DNA Mini Kit (Qiagen). Multiplex ligation-dependent probe amplification (MLPA) was used to determine copy-number alteration (CNA) of *EGFR*, *PTEN*, *CDKN2A*, *PDGFRA*, *CDK4*, *MDM2*, *NFKBIA*, and *TP53* (SALSA MLPA KIT probemix P105-D2, MRC-Holland, Amsterdam, the Netherlands). Sanger sequencing was conducted to assess mutations in telomerase reverse transcriptase promoter (*TERT*p) and to exclude mutations in *BRAF*, *H3F3A*, *IDH1/2,* and *HIST1H3B.* The *IDH1/2* mutation status, including non-canonical variants, was also evaluated.

### Statistical Analysis

We used the Wilcoxon rank-sum test for non-paired samples and the Wilcoxon signed-rank test for paired samples to compare diagnostic values between the 2 groups. Survival analyses were evaluated using the log-rank test. In this study, we particularly assessed 2 types of survivals outcomes. The first is OS, calculated from the day of surgery until death or the last follow-up. The second is radiological tumor overall survival (rOS), measured from the time of radiological birth until death or the last follow-up. The OS represents the typical OS used elsewhere, indicating the survival of GB patients after surgery. In contrast, rOS reflects survival from its radiological birth. Kaplan-Meier curve analyses were performed to evaluate survival outcomes based on age at diagnosis (≥65 and <65 years old), MIB-1 labeling index (≥30% and <30%), and molecular alterations, including CNA in *EGFR*, *PTEN*, and *CDKN2A*, or mutation in *TERT*p. The statistical analyses were performed using R version 4.2.3 (https://www.r-project.org/) and Prism (GraphPad). The data generated in this study are available within the article and its supplementary data files.

## Results

**Figure 1.**
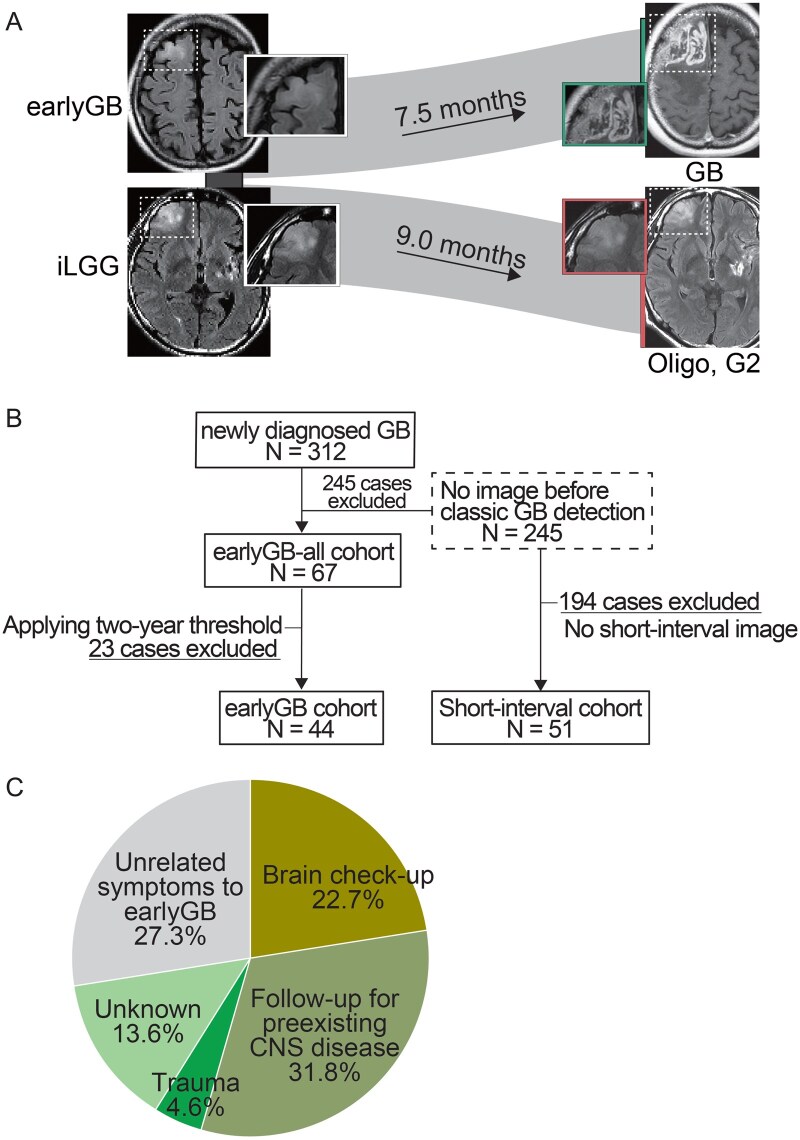
(A) A Sankey diagram illustrating the radiological changes. At the early stages of detection, both incidental low-grade glioma (iLGG) and early-stage glioblastoma (earlyGB) exhibit similar radiological features. It remains uncertain whether these lesions will remain as LGG or eventually progress to GB. (B) Flowchart of patient selection. (C) A Pie chart illustrating the reasons for undergoing radiological examinations that led to the detection of earlyGB.

Figure 1A illustrates that at the early stage of detection, gliomas exhibit a similar radiological appearance, regardless of whether they are classified as iLGG or earlyGB. However, it remains uncertain whether these lesions will remain as LGG or eventually progress to GB.

All cases included in the analysis were histologically confirmed as GB. To ensure a clear definition of classicGB, we only included cases with GB histology and excluded cases that met WHO 2021 molecular criteria for GB but lacked histological features of GB. Among the histologically confirmed GB cases, those harboring mutations in *IDH1/2*, *BRAF*, *H3F3A*, or *HIST1H3B* were excluded based on molecular testing.

Patient selection is illustrated in the flowchart (Figure 1B). Among the 312 patients, 245 cases did not have images before classicGB detection. The remaining 67 cases with available earlier imaging were classified as the earlyGB-all cohort. After applying the 2-year threshold, 23 cases were excluded, leaving 44 cases in the earlyGB cohort. In parallel, among the 245 cases without early imaging, 51 cases had 2 or more MRI scans after classicGB detection and were therefore classified into the short-interval cohort.

### Patient Characteristics of earlyGB Cohort

A total of 44 cases were included in the earlyGB cohort (Supplementary Tables S2 and S3). Radiological examinations leading to earlyGB detection were diverse: brain checkups in 10 cases (22.7%); follow-up for pre-existing central nervous system diseases in 14 cases (31.8%), including 9 with cerebrovascular disease, 3 with brain metastases, 1 with meningioma, and 1 with cavernous malformation; head trauma in 2 cases (4.6%); unknown reasons in 6 cases (13.6%); and unrelated symptoms to earlyGB in 12 cases such as headache, vertigo, and nausea (27.3%) (Figure 1C).

**Figure 2.**
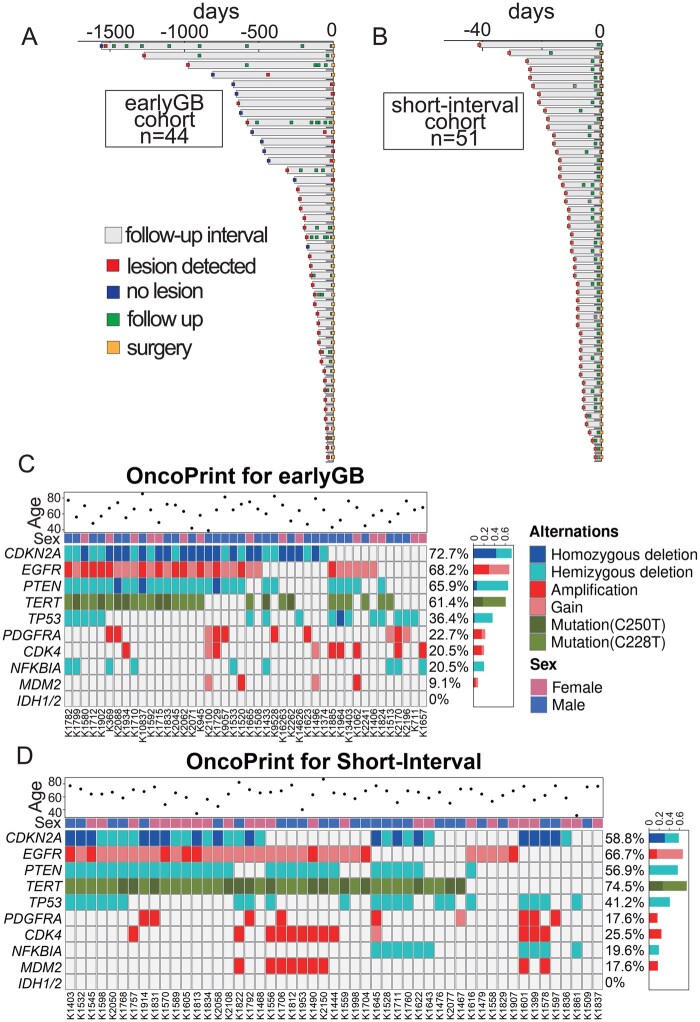
(A) and (B) Swimmer plots illustrate the follow-up in the earlyGB cohort (A) and short-interval cohort (B), including the timeline from initial radiological examination to surgery. Key events such as lesion detection, no lesion, and follow-up scans are shown. (C) and (D) OncoPrints for the earlyGB (C) and short-interval (D) cohorts, showing major molecular alterations in GB, including *CDKN2A* hemi/homozygous deletion, *EGFR* gain/amplification, *PTEN* hemi/homozygous deletion, and *TERT*p mutation. No significant difference in these variables was observed between the 2 cohorts.

The median interval between the earlyGB and the classicGB images was 155 days (range: 35-1,557 days), as illustrated in the swimmer plot (Figure 2A). The case with the longest interval (Case #: K2196), shown in Supplementary Figure S1, required 52 months to radiologically evolve into classicGB. The median interval between classicGB image and surgery was 3 days (mean: 5.6 days, range: 1-50 days, Supplementary Table S2). The median age at GB diagnosis was 60 years, with 29 patients (65.9%) being male. The mean tumor volume at the classicGB detection was 28.8 ± 23.8 cm^3^, and in 11 cases, no measurable tumor was observed at the time of earlyGB imaging. In total, 18 patients (40.9%) underwent more than 3 radiological examinations prior to surgery, and 6 cases were evaluated by CT during the early phase.

### Patient Characteristics of the Short-Interval Cohort

The short-interval cohort included 51 cases (Supplementary Table S2). The median interval between the 2 treatment-naïve preoperative radiological images was 8 days (range: 1-41 days), as illustrated in the swimmer plot (Figure 2B). The median interval between classicGB image and surgery was 8 days (mean: 11 days, range: 2-42 days). The median age at diagnosis was 65 years, with 26 patients (51.0%) being male. The mean tumor volume at the classicGB detection was 43.7 ± 34.5 cm^3^.

### Molecular Data in the earlyGB Cohort and Short-Interval Cohort

In the earlyGB cohort, mean MIB1 LI was 33.0 ± 13.6%, 27 cases (61.4%) had *TERT*p mutation, with 19 cases harboring C228T and eight cases C250T. 30 cases (68.2%) had *EGFR* gain/amplification, 29 cases (65.9%) had *PTEN* hemi/homozygous deletion, and 32 cases (72.7%) had *CDKN2A* hemi/homozygous deletion (Supplementary Table S2 and Figure 2C).

In the short-interval cohort, the mean MIB1 LI was 32.9 ± 12.8%, 38 cases (74.5%) had *TERT*p mutation, with 24 cases harboring C228T and 14 cases C250T. 34 cases (66.7%) had *EGFR* gain/amplification, 29 cases (56.9%) had *PTEN* hemi/homozygous deletion, and 30 cases (58.8%) had *CDKN2A* deletion (Figure 2D). No significant difference was found in genetic features between the earlyGB cohort and the short-interval cohort (Supplementary Table S2 and ­Figure 2C and D).

### Estimated Radiological Birth of GB

**Figure 3.**
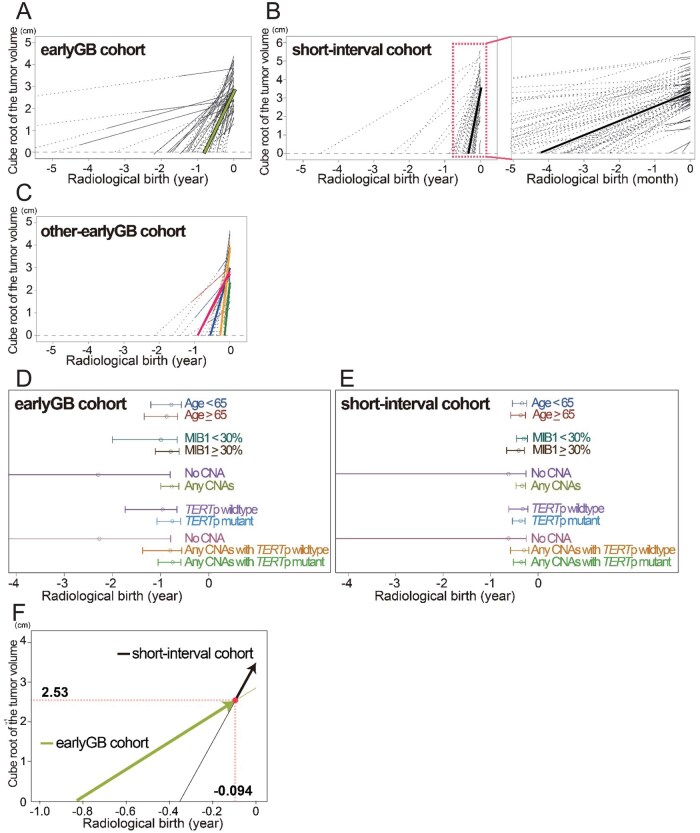
(A) and (B) Radiological birth of the earlyGB (A) and short-interval (B) cohorts, calculated using a linear radial growth model. The thin solid lines represent data from actual measurements, and the thin dotted lines represent extrapolated data from mathematical estimations (A-C). The thick solid lines indicate the average growth model (A and B). In the short-interval cohort (B), a magnified image highlights the narrow interval range. (C) Radiological birth of other-earlyGB cohort, also calculated using a linear radial growth model. The solid lines represent actual data ranges, and the dotted lines represent extrapolated data. Each color indicates a different study, and thick lines indicate average growth models. (D) and (E) Estimated radiological birth of earlyGB and short-interval cohorts stratified by key variables: age (<65 vs ≥65 years), MIB-1 labeling index (<30% vs ≥30%), copy-number alterations (CNA) in *EGFR*, *PTEN*, and *CDKN2A* (none vs any), *TERT*p mutation (mutant vs wildtype), and combined molecular subgroups. (F) The average growth trajectories of the earlyGB and short-interval cohorts were superimposed on a single coordinate axis, revealing an intersection point that indicates an abrupt change in growth slope.

We selected the linear radial growth model as the final, more robust model for the following 3 reasons. First, the exponential growth model produced clear outliers in earlyGB cohort (Supplementary Figure S2A and B). Second, the linear radial growth model provided more reasonable estimates of “radiological birth” compared with the exponential growth model (Supplementary Figure S2C and D and Supplementary Table S4). Third, the linear radial growth model achieved higher conditional *R*^2^ values—0.582 for the earlyGB cohort and 0.976 for the short-interval cohort, representing the proportion of variance explained by both fixed and random factors—compared with 0.526 and 0.818, respectively, for the exponential growth model (Supplementary Table S4). The results of mathematical analyses using the linear radial growth model across earlyGB cohort, short-interval cohort, and other-earlyGB cohort were demonstrated in Figure 3A-E. For interpretation, the radiological birth reported below represents the typical value predicted by the model from the fixed effects rather than an average of the patient-specific estimates, and negative values indicate time before symptom onset.

#### earlyGB cohort

Mathematical analysis using earlyGB cohort demonstrated that the radiological birth of GB was 0.83 years (95% CI: −1.10 to −0.66) before the symptomatic phase (Figure 3A and D and Table 1). For patients younger than 65 years (<65-year-old), the estimated radiological birth was −0.77 years (95% CI: −1.20 to −0.57), compared to −0.87 years (95% CI: −1.34 to −0.65) for those aged 65 years and older (≥65-year-old). For patients with a high MIB1 LI (≥30%), the estimated radiological birth was −0.79 years (95% CI: −1.11 to −0.61), compared to −0.99 year (95% CI: −2.00 to −0.66) for low MIB1 LI (< 30%). Similarly, −0.76 years (95% CI: −1.08 to −0.58) in *TERT*p mutation and −0.96 years (95% CI: −1.74 to −0.66) in *TERT*p wildtype. Cases with at least one CNA in either *EGFR*, *PTEN*, or *CDKN2A* exhibited a shorter time to radiological birth of −0.80 years (95% CI: −1.37 to −0.56) than no CNA of −2.27 years (95% CI: −100 to −0.79). Cases with at least one CNA in *EGFR*, *PTEN*, and *CDKN2A* along with *TERT*p mutation showed the earliest radiological birth at −0.75 years (95% CI: −1.05 to −0.58), whereas tumors without any of the 4 alterations presented a slow progression at −2.27 years (95% CI: −100 to −0.79) (Figure 3A and D, and Table 1).

**Table 1.** Radiological birth of earlyGB cohort and short-interval cohort

		earlyGB cohort				Short-interval cohort				Other-earlyGB cohort	
		*N*	Radiological birth, years (95% CI)	Slope, cm	*P*-value	*N*	Radiological birth, years (95% CI)	Slope, cm	*P*-value	*N*	Radiological birth, years
all		44	−0.83 (−1.10, −0.66)	3.44		51	**−**0.35 (−0.49, −0.27)	9.89		23	−0.92∼−0.15
Age at diagnosis	<65 y old	20	−0.77 (−1.20, −0.57)	3.69	.64	23	−0.34 (−0.54, −0.24)	10.41	.73		
	≥65 y old	24	−0.87 (−1.34, −0.65)	3.27		28	−0.37 (−0.58, −0.27)	9.51			
MIB-1 labeling index	<30%	15	−0.99 (−2.00, −0.66)	2.92	.44	21	−0.30 (−0.45, −0.22)	11.62	.25		
	≥30%	28	−0.79 (−1.11, −0.61)	3.65		30	−0.41 (−0.66, −0.29)	8.62			
At least one CNA in *EGFR, PTEN or CDKN2A*	No	5	−2.30 (−100, −0.80)	1.24	.063	6	−0.62 (-100, −0.25)	5.62	.28		
	Yes	39	−0.77 (−1.00, −0.62)	3.73		45	−0.34 (−0.47, −0.26)	10.36			
*TERT*p mutation	No	17	−0.96 (−1.74, −0.66)	2.98	.38	13	−0.32 (−0.62, −0.21)	10.87	.66		
	Yes	27	−0.76 (−1.08, −0.58)	3.77		38	−0.37 (−0.54, −0.27)	9.57			
At least one CNA in *EGFR, PTEN* or *CDKN2A* with or without *TERT*p mutation	No CNA with or without *TERT*p mutation	5	−2.27 (−100, −0.79)	1.26	.18	6	−0.62 (−100, −0.25)	5..64	.48		
	At least one CNA without *TERT*p mutation	13	−0.80 (−1.37, −0.56)	3.58		10	−0.29 (−0.58, −0.20)	11.84			
	At least one CNA with *TERT*p mutation	26	−0.75 (−1.05, −0.58)	3.83		35	−0.35 (−0.52, −0.26)	9.95			

Abbreviation: CNA, copy number alteration.

For reference, earlyGB-all cohort (*N* = 66), which includes all earlyGB cases without 2-year thresholds, demonstrated that the radiological birth of GB was −2.58 years (95% CI: −3.39 to −2.08) using the linear radial growth model and −2.58 years (95% CI: −3.30 to −1.92) using the exponential growth model before the symptomatic phase (Supplementary Table S5).

#### Short-interval cohort

In a similar analysis to the earlyGB cohort, the short-interval cohort demonstrated that the radiological birth of GB was 0.35 years prior to the symptomatic phase (95% CI: −0.49 to −0.27) (Figure 3B and Table 1). The estimated radiological birth of GB was −0.34 years (95% CI: −0.54 to −0.24) in patients younger than 65 years (< 65-year-old), and −0.37 years (95% CI: −0.58 to −0.27) in those 65 years and older (≥65-year-old). For tumors with a high MIB1 LI (≥ 30%), the radiological birth was −0.41 years (95% CI: −0.66 to −0.29) and for those with a low MIB1 LI (< 30%) was −0.30 years (95% CI: −0.45 to −0.22). Similarly, the radiological birth was −0.37 years (95% CI: −0.54 to −0.27) in *TERT*p mutation and −0.32 years (95% CI: −0.62 to −0.21) in *TERT*p wildtype. Tumors with at least one CNA in *EGFR*, *PTEN*, and *CDKN2A* along with a *TERT*p mutation showed the radiological birth of −0.35 years (95% CI: −0.52 to −0.26) and those without any of the 4 alterations presented the birth of −0.62 year (95% CI: −100 to −0.25). None of the factors analyzed showed a statistically significant difference in the duration of radiological birth within the short-interval cohort.

#### Other-earlyGB cohort

To validate our earlyGB cohort, we assessed other-earlyGB cohort as analyzed using earlyGB cohort and short-interval cohort. This other-earlyGB cohort indicated that the radiological birth of the GB was between −0.92 and −0.15 years before the symptomatic phase (Table 2. and Figure 3C: Red, Altieri et al. Blue, Ceravolo et al. Yellow, Simonet et al. and Green, Faguer et al.). This cohort did not include data on immunohistochemical, histopathological, or genetic analyses, preventing further analyses.

**Table 2.** Radiological birth of other-earlyGB cohort

		*N*	Slope, cm	Radiological birth, year	95% CI lower	95% CI upper
Our earlyGB cohort		44	3.44	−0.83	−1.10	−0.66
Our short-interval cohort		51	9.89	−0.35	−0.49	−0.27
Other-earlyGB cohort	Altieri et al	3	2.97	−0.92		−0.27
	Ceravolo et al	11	5.33	−0.56	−27.1	−0.30
	Faguer et al	4	15.17	−0.15	−0.34	−0.09
	Simonet et al	5	14.09	−0.28	−0.46	−0.20

#### Intersection between earlyGB and short-interval cohorts

Next, we superimposed the 2 trajectories of the earlyGB and short-interval GB cohorts on a single coordinate axis based on the *Y* = 0 and slope data and identified an intersection point (Figure 3F). Notably, the 2 trajectories met at a distinct angle, indicating a clear change in slope between the lines.

### OS and rOS in earlyGB Cohort

In our earlyGB-cohort, the median OS was 20.5 months (1.7 years), and OS based on analyzed molecular variables were shown in Supplementary Table S6.

**Figure 4.**
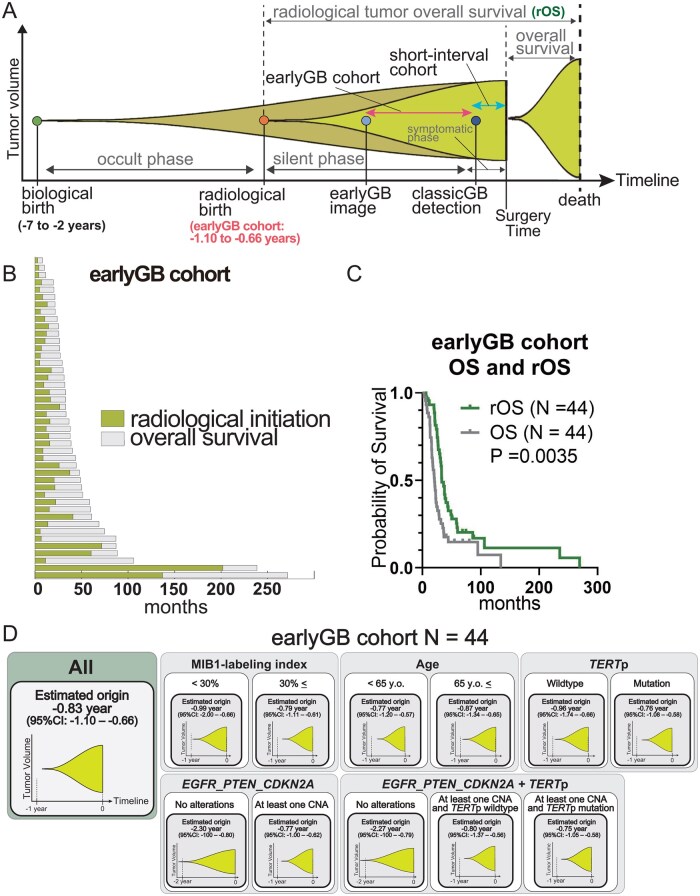
(A) A visual illustration of the overall timeline in glioblastoma, starting from biological birth (estimated 2 to 7 years before diagnosis), followed by the occult phase, radiological birth (estimated 0.66 to 1.10 years in earlyGB cohort), silent phase, symptomatic phase, detection of earlyGB, detection of classicGB, and surgical intervention. The period from radiological birth to death is defined as radiological tumor overall survival (rOS). (B) An overview of rOS in the earlyGB cohort, distinguishing radiological birth and OS with different color indicators. (C) Kaplan-Meier analyses of rOS and OS in the earlyGB cohort (*P* = .0035). (D) A visual summary of the estimated radiological birth in the earlyGB cohort. Each panel corresponds to a subgroup defined by clinical or molecular variables, displaying the estimated time of radiological birth with 95% confidence intervals. The horizonal axis denotes the time in years prior to surgery (time zero), and the vertical axis represents tumor volume.

Next, we conducted the analyses of rOS (Figure 4A-C). Illustrations of the overall timeline from biological birth, occult phase, radiological birth, silent phase, symptomatic phase, earlyGB image, classicGB detection, death, rOS, and OS were shown in Figure 4A. Also, the swimmer plot of rOS, the combination of radiological initiation and OS, was shown in Figure 4B. The median rOS was 33.2 months (Supplementary Table S7) or 2.8 years, longer than OS (Figure 4C). rOS based on analyzed molecular variables were shown in Supplementary Table S7. The factors related to shorter rOS were *TERT*p mutation (*P* = .081), CNA in *CDKN2A* (*P* = .0009), and at least one CNA in *EGFR*, *PTEN*, or *CDKN2A* (*P* = .071). The schematic illustration of our study based on MIB1-LI, Age, *TERT*p status, and the status of *EGFR*, *PTEN*, and *CDKN2A* was shown in Figure 4D.

## Discussion

In this study, we utilized earlyGB data to estimate the transition from occult phase to silent phase, representing the radiological birth of GB. We employed 2 distinct approaches: the earlyGB cohort, comprising rare clinical cases with longitudinal imaging, and the short-interval cohort, a dataset commonly used in prior studies to estimate tumor growth dynamics.^8^^,^^21^^,^^22^^,^^24^^,^^26-28^^,^^32^ We discuss our findings from 3 perspectives: the radiological birth of GB, the mathematical models of tumor growth, and the timeline from radiological birth to death.

### Estimated Radiological Birth of GB among Different Cohorts

Both our earlyGB and other-earlyGB cohorts suggested that the radiological birth of GB occurred before the symptomatic phase, with estimates of −0.83 years (95% CI: −1.10 to −0.66) in our cohort and −0.92 to −0.15 years in other-earlyGB cohort.^8^^,^^21^^,^^23^^,^^29^   The precise timing of the radiological birth may vary due to differences in tumor volume at classicGB detection and the interval length between earlyGB and classicGB imaging. In our earlyGB cohort, the range of tumor volume at the classicGB detection was 2.87-94.2 mL, comparable to tumor volume reported in other studies: 0.05-146.5^24^ and 0.1-83.2 mL.^39^ These findings indicate that tumor volume did not significantly differ across studies, supporting the robustness of our earlyGB cohort. Regarding interval lengths, the shortest and median intervals between earlyGB and classicGB imaging of our earlyGB cohort were 35 days and 5.2 months, respectively. Previous studies have described extremely short intervals, including 3 days, 4 days, 6 days,^8^ 3 weeks,^8^^,^^19^ 23 days,^27^ or 1-1.5 months.^6^ The inclusion of these short intervals likely shifts the estimated radiological birth closer to the time of classicGB detection. However, excluding such extreme cases, most reported intervals fall within similar ranges: 2-12 months,^8^ 10.3 months,^20^ 3.5-4 months,^31^ 2.4-5.9 months,^5^ 2-8 months,^14^^,^^18^ and 2-13 months.^6^ The consistency in interval lengths among studies, including our own, supports the general applicability of our radiological birth estimates.

We next explored the biological implications of analyzing the 2 cohorts together. When the trajectories of the earlyGB and short-interval cohorts were superimposed on a single coordinate axis, they intersect at a single point. The earlyGB cohort captures the non-enhancing, early phase of radiological development, whereas the short-interval cohort reflects the later, more aggressive phase of radiological development. Thus, this intersection likely represents a transition from one growth phase to another, rather than a mathematical coincidence.

One important question is why some non-enhancing earlyGB lesions dramatically transform into classicGB within as little as 3 days.^8^ Neither earlyGB nor short-interval cohort alone can fully explain this abrupt change. In this context, the intersection we identified corresponds to a radiological transformation point at which the growth slope changes abruptly, accounting for the rapid shift observed on interval imaging. Indeed, if one examines an earlyGB image obtained just before—and a classicGB image just after—this transformation point, the rapid change vividly illustrates the rapid radiological transition. Consequently, our analysis not only estimates the radiological birth of GB but also provides a dynamic model for understanding the radiographic evolution of GB.

### Estimated Radiological Birth of GB Varied by Molecular Alterations

Fan et al. investigated the relationship between molecular features and radiological tumor growth in WHO grade III and IV glioma (WHO 2016 classification), including both *IDH1*-mutant and wildtype cases.^40^ They found that *TERT*p mutation and high MIB1 LI were associated with increased tumor growth.^40^ Similarly, our study suggests that *TERT*p mutation and high MIB1 LI correlate with a later radiological birth compared to cases with *TERT*p wildtype and low MIB1 LI.

Korber et al. proposed a model suggesting that alterations in *EGFR*, *PTEN*, or *CDKN2A* occur early in tumor evolution, with *TERT*p mutation emerging later and driving rapid tumor growth.^2^ In contrast, Appin et al.reported that *TERT*p mutation is a clonal, early event in glioma evolution based on 3-dimensional maximal tumor sampling combined with deep sequencing.^41^ Our findings partly support Korber’s model, as CNAs in *EGFR*, *PTEN*, or *CDKN2A* were associated with steeper tumor growth. Although additional *TERT*p mutations appeared to further accelerate this growth, the effect was not robust. If *TERT*p mutation is indeed an early clonal event, it may not drive rapid tumor growth, which is consistent with the trends observed in our findings. Therefore, the precise role of *TERT*p mutation in GB progression, and its influence on tumor growth dynamics, warrants further investigation.

#### Statistics and mathematical model of GB growth

Mathematical growth models of LGG^42^^,^^43^ and meningiomas^44^ can be assessed using sequential radiological studies due to their slow-growing nature, which typically does not require urgent surgical intervention. In contrast, GB, a fast-growing malignant tumor, requires early surgical intervention, making sequential radiological follow-up less feasible. As a result, statistical and mathematical estimations of GB growth often rely on limited data, primarily short-interval MRI scans obtained before surgery. To address this limitation, 3 mathematical methods (linear radial, exponential, and Gompertzian growth models) have been applied to estimate the later phase of tumor growth.^24^ However, the reliability of such short-interval datasets for estimating the radiological birth of GB has not been adequately discussed. Estimating the radiological birth from the short-interval cohort statistically requires long-term retrograde extrapolations, which involves predicting data points beyond the measurable range and may introduce inaccuracies. Consequently, these estimations are primarily based on extrapolated data, thereby reducing their reliability.

In contrast, our study focuses on the retrograde estimation of radiological birth using longitudinal earlyGB data, which minimizes extrapolations and improves biological plausibility. Prior work indicated that in the early phase, tumor growth remains consistent among the 3 models except for very small tumors (<1 mL).^24^ Although some degree of extrapolation is still necessary for earlyGB cohort, the extent is considerably smaller, resulting in a more reliable dataset that revealed differences across clinical and molecular variables. This makes the earlyGB cohort more suitable for investigating the radiological evolution of GB and highlights the importance of utilizing datasets with minimal extrapolation to ensure more accurate insights into the early development of GB.

In our mathematical model, we applied a 2-year threshold to define the earlyGB cohort. Radiologically, even *de novo* GB passes through a non-enhancing phase before evolving into classicGB; this early phase likely exists in all cases but is rarely captured clinically and typically detected by chance. In our expanded earyGB-all cohort, including all available earlyGB images without any threshold, yielded an estimated radiological birth of −2.58 years (95% CI: −3.39 to −2.08). However, because most published earlyGB cases fall within approximately 1 year (Supplementary Table S1), this extended estimate likely does not reflect real-world data. Prior evidence indicates that genetic alterations precede radiological detection in both GB^2^ and medulloblastoma,^45^^,^^46^ suggesting a biological lag between molecular initiation and radiological manifestation. Therefore, we selected a 2-year threshold based on previous genetic studies demonstrating that the biological birth of GB occurs 2-7 years before diagnosis.^2^ Using the broader 2-7 years range could create an overlap period during which genetic alterations are already present despite an absence of radiological detectable lesions. Although applying this threshold reduced the number of analyzable cases from 66 in earlyGB-all cohort to 44 in earlyGB cohort, the resulting dataset more accurately reflects the radiological biology of GB.

#### rOS of GB

The optimal management of iLGG, particularly whether to intervene early or adopt a watch-and-wait approach, remains a highly debated topic.^9^^,^^11^ Evidence suggests that early surgical intervention may significantly prolong OS by preventing malignant transformation.^9^^,^^47^ Notably, both Jakola et al. and Ius et al.demonstrated the clinical importance of early surgery for iLGG, irrespective of *IDH* status.^9^^,^^11^ iLGG, defined as uniformly non-enhancing lesion, includes 14.7%-31% of *IDH*-wildtype cases,^9-11^ indicating that a subset of such non-enhancing lesions likely represents earlyGB, which would ultimately be classified as GB under the WHO 2021 classification. Understanding the clinical course of lesions that eventually progress to GB is therefore crucial, and our rOS data provide insight into the natural clinical course of these non-enhancing lesions.

Traditionally, OS is defined as the period between surgical intervention and death. In contrast, the present study enabled us to examine the entire timeline of GB from radiological birth to death, which we refer to as rOS. We identified a median rOS of 33.2 months or approximately 2.8 years. This rOS data represent the natural course of GB when surgical intervention is performed only after classicGB appeared. In the early phase, when FLAIR abnormalities are detected, it is challenging to determine whether a non-enhancing lesion will progress to GB or remain as LGG (Figure 1A). Our findings suggest that if such a lesion represents an ealryGB, the expected remaining lifespan may be approximately 2.8 years—underscoring the clinical significance of recognizing this phase and potentially supporting earlier surgical intervention. Accordingly, our analysis of earlyGB offers a new perspective for optimizing management strategies for iLGG. Nevertheless, deciding on early surgical intervention during the asymptomatic phase remains difficult because of the significant imaging overlap between iLGG and earlyGB. Differentiating these entities based solely on conventional MRI is particularly challenging in the early phase. Future research using radiomics or other advanced imaging approaches should aim to elucidate whether these imaging abnormalities represent iLGG or earlyGB.

In our study, molecular alterations in earlyGB cohort were determined from classicGB specimens and may not precisely reflect the alterations at the early phase. Although not asymptomatic, *IDH*-wildtype grade II and III gliomas harbored *TERT*p mutations and CNA in *EGFR*, *PTEN*, and *CDKN2A*.^48^ Our findings indicate that the presence of at least one CNA in these genes is associated with earlier progression. Therefore, early molecular profiling through biopsy or radiomics in asymptomatic lesions may clarify the clinical timeline and guide decisions on aggressive surgical intervention.

### Limitation

This study has several limitations. First, the retrospective nature and the rarity of earlyGB images hold selection bias. Because earlyGB images were collected from multiple institutions over several years, the available imaging modalities were heterogeneous, and a single case was not consistently analyzed using the same sequence. In addition, interobserver variability in manual tumor segmentation may exist. Nonetheless, we believe that such variability and heterogeneity are unlikely to substantially alter the overall concept or conclusions of this study. According to data from the Organization for Economic Co-operation and Development in 2020, the number of MRI units per 1 million inhabitants is 57.39 in Japan, followed by 35.02 in Germany, 34.66 in United States, 34.24 in Korea. Although earlyGB is a rare phenomenon, the high medical accessibility in Japan facilitated the identification of earlyGB cases and enabled this study. Second, the intervals between earlyGB and classicGB varied across cases. While previous studies have reported intervals as short as 3 days,^5^^,^^6^^,^^8^^,^^14-16^^,^^18-20^^,^^23^^,^^31^ our earlyGB cohort had the shortest interval of 35 days, indicating that very fast-growing GB cases were not included. Therefore, inclusion of very fast-growing cases may estimate the radiological birth shorter. Third, we primarily focused on GB s with typical histological and radiological features; therefore, cases that were not histologically diagnosed as GB but met the WHO 2021 molecular criteria for GB were excluded. Finally, we discussed earlyGB and iLGG in the context of early surgical interventions. Differentiating between T2/FLAIR abnormalities that will progress to GB (earlyGB) and those that will remain as iLGG remains clinically challenging. Although we proposed a clinical timeline of approximately 2.8 years from radiological birth to death, this estimate is derived from patients who ultimately developed GB. Therefore, it cannot be applied to all MRI abnormalities, as some T2/FLAIR lesions represent non-neoplastic lesions. Further research focused on radiological markers in the early phase is essential to improve diagnostic accuracy and refine treatment decision-making for these lesions.

### Conclusion

This study defines the radiological biology of GB, illustrating a continuous evolution from the silent to the symptomatic phase. Integration of the earlyGB and short-interval cohorts revealed an intersection point between their growth trajectories, representing a radiological transformation stage where tumor growth accelerates. Molecular alterations, particularly the presence of CNA in *EGFR*, *PTEN*, or *CDKN2A*, were found to accelerate tumor growth. The median interval from radiological birth to death of GB was estimated at 2.8 years. Collectively, these findings provide valuable insights into the biological and radiological progression of GB and may inform decision-making regarding early surgical intervention for clinically silent, non-enhancing lesions.

## Supplementary Material

vdaf275_Supplementary_Data

## Data Availability

Data will be made available upon reasonable request by contacting the corresponding author.
