# Supplementary material for: Retrograde longitudinal imaging analyses of IDH-wildtype glioblastoma reveal its clinical timeline from radiological birth to death
Source: Neurooncol Adv. 2026 Jan 2;8(1):vdaf275. doi: 10.1093/noajnl/vdaf275 (PMC12924880; doi:10.1093/noajnl/vdaf275)
Supplement: vdaf275_Supplementary_Data [file vdaf275_supplementary_data.zip › Supplementary_Table_and_Figure_Legends_2ndclean.docx]

**Supplementary Table 1. Previous studies of early-stage gliomas**

| Authors | Year | Included patients | N | Imaging modality | Interval between first radiological procedure and last one |
| --- | --- | --- | --- | --- | --- |
| Bolender | 1983 | GB | 8 | CT | 2 - 9 months |
| Blankenberg | 1995 | LGG or HGG or GB | 5 | CT or MRI | 186 – 1112 days |
| Landy | 2000 | HGG | 9 | MRI | 1 - 8 months |
| Okamoto | 2002 | GB or AA | 5 | MRI | 4 months - 3 years and 3months |
| Cohen-Gadol | 2004 | GB | 2 | MRI | 16 and 17weeks |
| Jung & Jung | 2007 | GB | 2 | MRI | 6 months |
| Gömöri | 2009 | GB | 1 | MRI | 1 month |
| Nishi | 2009 | HGG | 7 | MRI | 3 - 24 months |
| Oyama | 2010 | GB | 1 | MRI | 10 months |
| Chittiboina | 2012 | GB | 1 | MRI | 84 days |
| Simonet Redondo | 2012 | GB or AO | 6 | MRI | 28 - 179 days |
| Thaler | 2012 | GB | 15 | MRI | 0.7 - 76.6 months |
| Faguer | 2014 | GB | 3 | MRI | 1. - 2.0 months |
| Ideguchi | 2015 | GB | 5 | MRI | 2.4 - 5.9 months |
| Cochereau | 2016 | GB | 1 | MRI | 5 years and 6 months |
| Zhang | 2016 | GB | 1 | MRI | 23 days |
| Toh | 2017 | GB | 26 | MRI | 1 - 13 months |
| Altieri | 2020 | GB or AA | 4 | MRI | 61 - 742 days |
| Wang | 2020 | GB | 8 | CT and MRI | 4 – 15weeks |
| Ceravolo | 2021 | GB | 13 | MRI | 3 days – 12 months |
| Hakan | 2021 | GB | 2 | MRI | 3.5 and 4 months |
| Leclerc | 2024 | GB | 7 | MRI | >7 days |

GB, glioblastoma; LGG, low grade glioma; HGG, high grade glioma; AA, anaplastic astrocytoma; AO, anaplastic oligodendroglioma

**Supplementary Table 2. Clinical and molecular characteristics of patients in earlyGB cohort and short-interval cohort**

|  | **earlyGB cohort (n=44)** | **Short-interval cohort (n=51)** | P-value |
| --- | --- | --- | --- |
| Duration of radiological follow-up, day, median (range) | 155 (35-1557) | 9 (2-42) | <0.0001^a^ |
| Sex, male, n (%) | 29 (65.9) | 26 (51.0) | 0.15^b^ |
| Age at diagnosis, median (range) | 60 (39-85) | 65 (31-87) | 0.62^a^ |
| Tumor volume at the initial imaging, cm^3^, mean ± SD | 28.8 ± 23.8 | 43.7 ± 34.5 | <0.0001^a^ |
| MIB-1 labeling index, %, mean ± SD | 33.0 ± 13.6, Missing, 1 | 32.9 ± 12.8 | 0.62^a^ |
| *TERT*p mutation, n (%) | 27 (61.4) | 38 (74.5) | 0.19^b^ |
| Copy number alteration |  |  |  |
| *EGFR* amplification/gain, n (%) | 30 (68.2) | 34 (66.7) | 1.0^b^ |
| *PTEN* hemi/homozygous deletion, n (%) | 29 (65.9) | 29 (56.9) | 0.41^b^ |
| *CDKN2A* hemi/homozygous deletion, n (%) | 32 (72.7) | 30 (58.8) | 0.20^b^ |
| *PDGFRA* amplification/gain, n (%) | 10 (22.7) | 9 (17.6) | 0.61^b^ |
| *CDK4* amplification, n (%) | 9 (20.5) | 13 (25.5) | 0.63^b^ |
| *MDM2* amplification, n (%) | 4 (9.1) | 9 (17.6) | 0.25^b^ |
| *NFKBIA* hemizygous deletion, n (%) | 9 (20.5) | 10 (19.6) | 1^b^ |
| *TP53* hemizygous deletion, n (%) | 16 (36.4) | 21 (41.2) | 0.68^b^ |

a, Mann-Whitney test; b, Fisher’s exact test; GB, glioblastoma

**Supplementary Table 3. Clinical and molecular characteristics of all patients in earlyGB cohort**

| Patient | Age/sex | Reasons for previous radiological study | Duration of Radiological follow-up, days | Number of scans before surgery | Interval between classicGB detection and surgery, days | *IDH1/2* status | MIB1-LI |
| --- | --- | --- | --- | --- | --- | --- | --- |
| K369 | 67/F | Unknown | 60 | 2 | 8 | WT | NA |
| K711 | 65/F | Unknown | 577 | 9 | 18 | WT | 20% |
| K945 | 58/F | Unknown | 545 | 3 | 2 | WT | 40% |
| K1062 | 68/F | Unrelated symptoms | 974 | 5 | 50 | WT | 50% |
| K1374 | 65/M | Unrelated symptoms | 51 | 2 | 3 | WT | 10% |
| K1406 | 58/F | Unrelated symptoms | 124 | 4 | 1 | WT | 30% |
| K1433 | 60/M | Unrelated symptoms | 40 | 3 | 1 | WT | 15% |
| K1496 | 79/M | Unrelated symptoms | 53 | 2 | 2 | WT | 50% |
| K1508 | 66/M | Brain check-up | 434 | 2 | 12 | WT | 40% |
| K1513 | 50/M | Unknown | 621 | 2 | 1 | WT | 50% |
| K1520 | 72/M | Follow-up for preexisting CNS disease | 309 | 5 | 2 | WT | 20% |
| K1533 | 65/M | Brain check-up | 193 | 4 | 3 | WT | 50% |
| K1580 | 70/F | Unrelated symptoms | 100 | 2 | 2 | WT | 40% |
| K1592 | 65/F | Brain check-up | 158 | 2 | 4 | WT | 30% |
| K1623 | 47/M | Follow-up for preexisting CNS disease | 224 | 2 | 1 | WT | 20% |
| K1657 | 68/F | Unrelated symptoms | 56 | 2 | 3 | WT | 40% |
| K1665 | 75/F | Follow-up for preexisting CNS disease | 672 | 3 | 2 | WT | 30% |
| K1710 | 66/F | Brain check-up | 151 | 2 (initial image: CT) | 5 | WT | 30% |
| K1712 | 48/M | Brain check-up | 464 | 2 | 2 | WT | 18% |
| K1715 | 49/F | Brain check-up | 39 | 2 | 3 | WT | 30% |
| K1729 | 65/M | Brain check-up | 138 | 2 (initial image: CT) | 4 | WT | 30% |
| K1782 | 77/M | Follow-up for preexisting CNS disease | 480 | 2 | 3 | WT | 50% |
| K1799 | 56/M | Brain check-up | 42 | 2 | 2 | WT | 33% |
| K1824 | 64/F | Follow-up for preexisting CNS disease | 124 | 2 | 1 | WT | 35% |
| K1833 | 72/M | Follow-up for preexisting CNS disease | 79 | 2 (initial image: CT) | 17 | WT | 27% |
| K1885 | 43/M | Unknown | 259 | 2 (initial image: CT) | 4 | WT | 35% |
| K1902 | 57/M | Unrelated symptoms | 35 | 2 | 3 | WT | 18% |
| K1934 | 55/M | Unrelated symptoms | 85 | 4 | 9 | WT | 30% |
| K1964 | 52/M | Unrelated symptoms | 51 | 2 | 3 | WT | 10% |
| K2045 | 71/M | Follow-up for preexisting CNS disease | 238 | 3 | 5 | WT | 25% |
| K2062 | 63/F | Follow-up for preexisting CNS disease | 96 | 3 | 4 | WT | 40% |
| K2071 | 42/M | Follow-up for preexisting CNS disease | 41 | 2 | 7 | WT | 40% |
| K2088 | 74/M | Follow-up for preexisting CNS disease | 97 | 3 | 7 | WT | 65% |
| K2100 | 39/M | Unknown | 178 | 8 | 3 | WT | 25% |
| K2170 | 60/M | Brain check-up | 219 | 2 (initial image: CT) | 6 | WT | 60% |
| K2196 | 76/M | Follow-up for preexisting CNS disease | 1557 | 10 | 11 | WT | 30% |
| K2241 | 45/M | Trauma | 194 | 2 (initial image: CT) | 1 | WT | 30% |
| K9057 | 81/M | Follow-up for preexisting CNS disease | 55 | 3 | 3 | WT | 30% |
| K9528 | 82/M | Follow-up for preexisting CNS disease | 171 | 2 | 2 | WT | 50% |
| K16263 | 71/F | Unrelated symptoms | 148 | 3 | 9 | WT | 23% |
| K10837 | 85/M | Follow-up for preexisting CNS disease | 809 | 3 | 3 | WT | 15% |
| K13403 | 76/M | Unrelated symptoms | 1271 | 4 | 1 | WT | 23% |
| K14626 | 64/F | Trauma | 650 | 2 | 5 | WT | 60% |
| K2262 | 51/M | Brain check-up | 106 | 2 | 9 | WT | 20% |

CNS, central nervous system; WT, wildtype

| Patient | *TERT*p mutation | CNA | | | | | | | | |
| --- | --- | --- | --- | --- | --- | --- | --- | --- | --- | --- |
|  |  | *EGFR* amp/gain | *PTEN* homo/hemi | *CDKN2A* homo/hemi | *PDGFRA* amp/gain | *CDK4* amp/gain | *MDM2* amp/gain | *NFKBIA* homo/hemi | *TP53* homo/hemi | At least one CNA in *EGFR, PTEN or CDKN2A* |
| K369 | C228T mut | amp | hemi | homo | amp | intact | intact | hemi | intact | Yes |
| K711 | WT | intact | intact | intact | intact | intact | intact | intact | hemi | No |
| K945 | C228T mut | amp | hemi | homo | intact | intact | intact | intact | intact | Yes |
| K1062 | WT | gain | hemi | intact | intact | amp | amp | intact | intact | Yes |
| K1374 | WT | intact | intact | hemi | intact | intact | intact | intact | intact | Yes |
| K1406 | WT | gain | intact | intact | intact | intact | intact | intact | hemi | Yes |
| K1433 | C250T mut | intact | hemi | hemi | intact | intact | intact | hemi | hemi | Yes |
| K1496 | WT | intact | intact | homo | intact | gain | gain | intact | intact | Yes |
| K1508 | WT | gain | intact | homo | intact | intact | intact | intact | intact | Yes |
| K1513 | C228T mut | intact | intact | intact | gain | intact | intact | hemi | intact | No |
| K1520 | WT | amp | hemi | hemi | intact | intact | amp | intact | intact | Yes |
| K1533 | WT | gain | hemi | homo | intact | intact | intact | hemi | intact | Yes |
| K1580 | C228T mut | amp | hemi | homo | intact | intact | intact | intact | hemi | Yes |
| K1592 | C228T mut | gain | hemi | hemi | intact | intact | intact | intact | intact | Yes |
| K1623 | WT | intact | intact | hemi | amp | intact | intact | intact | intact | Yes |
| K1657 | WT | intact | intact | intact | intact | amp | intact | hemi | intact | No |
| K1665 | C228T mut | gain | intact | homo | intact | intact | intact | intact | hemi | Yes |
| K1710 | C228T mut | gain | hemi | hemi | intact | intact | intact | hemi | intact | Yes |
| K1712 | C228T mut | amp | hemi | hemi | intact | intact | intact | intact | hemi | Yes |
| K1715 | C250T mut | amp | hemi | homo | intact | intact | intact | intact | intact | Yes |
| K1729 | WT | amp | homo | homo | amp | amp | intact | intact | intact | Yes |
| K1782 | C228T mut | amp | hemi | hemi | intact | intact | intact | hemi | hemi | Yes |
| K1799 | C250T mut | gain | hemi | hemi | intact | intact | intact | hemi | hemi | Yes |
| K1824 | C228T mut | intact | hemi | intact | intact | intact | intact | intact | hemi | Yes |
| K1833 | C250T mut | gain | hemi | homo | intact | intact | intact | intact | intact | Yes |
| K1885 | C228T mut | amp | hemi | intact | intact | amp | intact | intact | hemi | Yes |
| K1902 | C250T mut | amp | hemi | hemi | intact | intact | intact | intact | hemi | Yes |
| K1934 | C250T mut | gain | hemi | homo | intact | amp | intact | intact | intact | Yes |
| K1964 | C228T mut | gain | hemi | intact | intact | amp | intact | intact | homo | Yes |
| K2045 | C228T mut | amp | hemi | hemi | intact | intact | intact | intact | intact | Yes |
| K2062 | C228T mut | amp | hemi | homo | intact | intact | intact | intact | intact | Yes |
| K2071 | C228T mut | gain | hemi | homo | intact | intact | intact | intact | intact | Yes |
| K2088 | C250T mut | gain | homo | homo | amp | intact | intact | intact | intact | Yes |
| K2100 | WT | gain | hemi | homo | gain | gain | gain | intact | intact | Yes |
| K2170 | WT | intact | intact | intact | amp | amp | intact | hemi | hemi | No |
| K2196 | WT | intact | intact | intact | gain | intact | intact | intact | hemi | No |
| K2241 | C228T mut | gain | intact | intact | intact | intact | intact | intact | intact | Yes |
| K9057 | WT | gain | hemi | hemi | amp | intact | intact | intact | intact | Yes |
| K9528 | WT | intact | hemi | hemi | amp | intact | intact | intact | intact | Yes |
| K16263 | C228T mut | intact | intact | homo | intact | intact | intact | intact | intact | Yes |
| K10837 | C228T mut | amp | homo | homo | intact | intact | intact | intact | intact | Yes |
| K13403 | C228T mut | gain | hemi | intact | intact | intact | intact | intact | hemi | Yes |
| K14626 | WT | intact | intact | homo | intact | intact | intact | intact | hemi | Yes |
| K2262 | C250T mut | intact | intact | homo | intact | intact | intact | intact | intact | Yes |

F, female; M, male; MIB-1 LI, MIB-1 labeling index; WT, wildtype; CNA, copy number alteration; amp, amplification; mut, mutation

**Supplementary Table 4 Range and mean of “radiological birth”, and marginal and conditional R^2^ by each model in each cohort**

| cohort | model | range of “radiological birth” (year) | mean “radiological birth” (year) | marginal R^2^ | conditional R^2^ |
| --- | --- | --- | --- | --- | --- |
| earlyGB | linear radial growth model | -16.6 to -0.23 | -0.83 (-1.10, -0.66) | 0.355 | 0.582 |
|  | exponential growth model | -174.9 to -0.50 | -1.49 (-2.03, -1.17) | 0.374 | 0.526 |
| short-interval | linear radial growth model | -4.5 to -0.02 | -0.35 (-0.49, -0.27) | 0.025 | 0.976 |
|  | exponential growth model | -63.0 to -0.02 | -0.67 (-5.86, -0.36) | 0.015 | 0.818 |

**Supplementary Table 5 Radiological birth of earlyGB-all cohort**

| **earlyGB-all cohort (N = 66)** | | |
| --- | --- | --- |
| model | linear radial growth model | exponential growth model |
| range of “radiological birth” (year) | -34.8 to -1.04 | -98.0 to -0.55 |
| mean “radiological birth” (year) | -2.58 (-3.39, -2.08) | -2.44 (-3.30, -1.92) |
| marginal R^2^ | 0.294 | 0.397 |
| conditional R^2^ | 0.425 | 0.458 |

**Supplementary Table 6. Clinical and genetic parameters affecting OS in earlyGB cohort**

|  | | | N | Overall survival | |
| --- | --- | --- | --- | --- | --- |
|  |  |  |  | Median months | P-value |
| All | |  | 44 | 20.5 |  |
| Age at diagnosis | | < 65 year-old | 20 | 21.5 | 0.86^a^ |
|  |  | ≧ 65 year-old | 24 | 20.2 |  |
| Surgical procedure | | Resection | 38 | 21.2 | 0.072^a^ |
|  |  | Biopsy | 6 | 16.8 |  |
| MIB-1 labeling index | | < 30% | 15 | 18.3 | 0.26^a^ |
|  |  | ≧ 30% | 28 | 22.2 |  |
| *TERT* promoter | | Mutant | 27 | 17.9 | 0.12^a^ |
|  |  | Wildtype | 17 | 23.0 |  |
| CNA | *EGFR* | amplification/gain | 30 | 19.6 | 0.56^a^ |
|  |  | retain | 14 | 24.2 |  |
|  | *PTEN* | hemi/homozygous deletion | 29 | 20.2 | 0.66^a^ |
|  |  | retain | 15 | 25.9 |  |
|  | *CDKN2A* | hemi/homozygous deletion | 32 | 18.3 | 0.0049^a^ |
|  |  | retain | 12 | 34.8 |  |
|  | *PDGFRA* | amplification/gain | 10 | 21.5 | 0.72^a^ |
|  |  | retain | 34 | 20.4 |  |
|  | *CDK4* | amplification | 9 | 31.6 | 0.077^a^ |
|  |  | retain | 35 | 19.6 |  |
|  | *MDM2* | amplification | 4 | 80.2 | 0.081^a^ |
|  |  | retain | 40 | 19.6 |  |
|  | *NFKBIA* | hemizygous deletion | 9 | 23.0 | 0.45^a^ |
|  |  | retain | 35 | 20.2 |  |
|  | *TP53* | hemizygous deletion | 16 | 28.9 | 0.25^a^ |
|  |  | retain | 28 | 19.6 |  |
|  | At least one CNA in *EGFR, PTEN or CDKN2A* | No | 5 | 36.7 | 0.16^a^ |
|  |  | Yes | 39 | 20.7 |  |
|  | All CNA in *EGFR, PTEN* and *CDKN2A* | No | 22 | 24.2 | 0.18^a^ |
|  |  | Yes | 23 | 19.6 |  |
| At least one CNA in *EGFR, PTEN* or *CDKN2A* with or without *TERT*p mutation | | No CNA with or without *TERT*p mutation | 5 | 36.7 | 0.21^a^ |
|  |  | At least one CNA without *TERT*p mutation | 13 | 22.5 |  |
|  |  | At least one CNA with *TERT*p mutation | 26 | 17.5 |  |

CNA, copy number alteration; a, log-rank test

**Supplementary Table 7. Clinical and genetic parameters affecting radiological tumor OS (rOS) in earlyGB cohort**

|  | | | N | Radiological tumor Overall survival | |
| --- | --- | --- | --- | --- | --- |
|  |  |  |  | Median months | P-value |
| All | |  | 44 | 33.2 | - |
| Age at diagnosis | | < 65 year-old | 20 | 32.4 | 0.32^a^ |
|  |  | ≧ 65 year-old | 24 | 37.7 |  |
| Surgical procedure | | Resection | 38 | 33.2 | 0.64^a^ |
|  |  | Biopsy | 6 | 38.4 |  |
| MIB-1 labeling index | | < 30% | 15 | 33.2 | 0.81^a^ |
|  |  | ≧ 30% | 28 | 34.6 |  |
| *TERT* promoter | | Mutant | 27 | 32.8 | 0.081^a^ |
|  |  | Wildtype | 17 | 37.7 |  |
| CNA | *EGFR* | amplification/gain | 30 | 33.2 | 0.52^a^ |
|  |  | retain | 14 | 35.2 |  |
|  | *PTEN* | hemi/homozygous deletion | 29 | 33.2 | 0.57^a^ |
|  |  | retain | 15 | 37.7 |  |
|  | *CDKN2A* | hemi/homozygous deletion | 32 | 31.9 | 0.0009^a^ |
|  |  | retain | 12 | 86.1 |  |
|  | *PDGFRA* | amplification/gain | 10 | 27.2 | 0.53^a^ |
|  |  | retain | 34 | 37.1 |  |
|  | *CDK4* | amplification | 9 | 40.0 | 0.20^a^ |
|  |  | retain | 35 | 32.8 |  |
|  | *MDM2* | amplification | 4 | 60.4 | 0.060^a^ |
|  |  | retain | 40 | 32.8 |  |
|  | *NFKBIA* | hemizygous deletion | 9 | 38.2 | 0.54^a^ |
|  |  | retain | 35 | 32.8 |  |
|  | *TP53* | hemizygous deletion | 16 | 46.7 | 0.18^a^ |
|  |  | retain | 28 | 31.9 |  |
|  | At least one CNA in *EGFR, PTEN or CDKN2A* | No | 5 | 235.6 | 0.071^a^ |
|  |  | Yes | 39 | 32.8 |  |
|  | All CNA in *EGFR, PTEN* and *CDKN2A* | No | 22 | 40.6 | 0.13^a^ |
|  |  | Yes | 23 | 33.2 |  |
| At least one CNA in *EGFR, PTEN* or *CDKN2A* with or without *TERT*p mutation | | No CNA with or without *TERT*p mutation | 5 | 235.6 | 0.11^a^ |
|  |  | At least one CNA without *TERT*p mutation | 13 | 37.7 |  |
|  |  | At least one CNA with *TERT*p mutation | 26 | 32.8 |  |

CNA, copy number alteration; a, log-rank test

**Supplementary Figure 1**

A post-surgical follow-up for a frontal base meningioma revealed an asymptomatic cerebellar lesion showing exceptionally slow tumor growth. A FLAIR abnormal lesion was first detected 10 months after the initial image, followed by T1Gd enhancement 13 months later. This lesion (indicated by white arrows) eventually progressed to a classic GB appearance, confirmed 52 months after the initial radiological imaging.

**Supplementary Figure 2**

Comparison of the linear radial growth model and the exponential growth model using representative cases #K2196 (A and B) and #K1062 (C and D) from the earlyGB cohort. In case #K2196, the exponential model (B) produced an outlier at smaller tumor volumes (black arrow), expanding the fitted curve downward, whereas the linear radial growth model (A) provided a stable fit. In case #K1062, both models fit the observed data reasonably well; however, the estimated radiological birth was −16.6 years in (C) versus −174.9 years in (D), indicating that the exponential model is less suitable for extrapolation of tumor origin.
